# Impact of NR4A3 on wound healing in chronic venous ulcers and its association with the PI3K/Akt signaling pathway

**DOI:** 10.3389/fphys.2026.1822383

**Published:** 2026-08-31

**Authors:** Yu Zhou, Xiaorong Zhan, Tong Qiao

**Affiliations:** 1 Department of Intervention Vascular, The Wujin Clinical College of Xuzhou Medical University, Changzhou, Jiangsu, China; 2 Jiangsu Key Laboratory of New Drug Research and Clinical Pharmacy, Xuzhou Medical University, Xuzhou, Jiangsu, China; 3 Department of Interventional Vascular, Wujin People’s Hospital Affiliated with Jiangsu University, Changzhou, Jiangsu, China; 4 Department of Vascular Surgery, Nanjing Drum Tower Hospital, Clinical College of Nanjing Medical University, Nanjing, Jiangsu, China

**Keywords:** cell migration, chronic venous ulcer, inflammatory response, NR4A3, PI3K/Akt pathway

## Abstract

**Background:**

To investigate the role of NR4A3 in chronic venous ulcer (VU) wound healing and to explore its potential regulatory mechanism involving the PI3K/Akt pathway.

**Methods:**

Differential expression and Kyoto Encyclopedia of Genes and Genomes (KEGG) enrichment analyses were performed using the GSE174661 dataset. DEGs were filtered by |log2FC| > 1 and adjusted P < 0.05, with KEGG significance set at P < 0.05. NR4A3 was identified as the core gene. NR4A3 knockdown and overexpression were established in HaCaT cells to evaluate proliferation, migration, and inflammatory cytokines. TNF-α was used to mimic the inflammatory microenvironment. Western blotting assessed phosphorylation of GSK3β, mTOR, PI3K, and Akt. PI3K/Akt agonist 740Y-P and inhibitor LY294002 were used in rescue experiments.

**Results:**

Bioinformatic analysis revealed that NR4A3 expression was markedly downregulated in chronic venous ulcer (VU) tissues relative to normal skin and ordinary acute wound tissues. Differentially expressed genes were significantly enriched in the PI3K/Akt signaling pathway. TNF-α stimulation significantly upregulated NR4A3 expression and increased phosphorylation of GSK3β and mTOR in HaCaT cells. In cultured HaCaT keratinocytes, NR4A3 knockdown suppressed cell proliferation and invasion, enhanced cell migration, and elevated the expression and secretion of pro-inflammatory cytokines (IL-6, IL-8, CXCL5), accompanied by reduced phosphorylation of PI3K and Akt. Conversely, NR4A3 overexpression promoted cell proliferation and invasion, restrained migration, and dampened inflammatory responses, while increasing PI3K/Akt phosphorylation. Treatment with the PI3K/Akt agonist 740Y-P partially rescued the impaired proliferation, aberrant migration, and excessive inflammation caused by NR4A3 silencing, whereas PI3K/Akt inhibitor LY294002 aggravated pathway suppression. These findings suggest that NR4A3-associated changes in keratinocyte functions and inflammatory reactions are functionally linked to PI3K/Akt pathway activity, and inflammatory stimulation activates GSK3β/mTOR signaling accompanied by compensatory NR4A3 upregulation.

**Conclusion:**

These findings suggest that NR4A3 is associated with keratinocyte behavior and inflammatory responses via the PI3K/Akt pathway, potentially affecting chronic VU progression and healing. Reduced NR4A3 may impair wound repair through inflammation and abnormal cell migration, while TNF-α induces compensatory NR4A3 elevation.

## Introduction

Chronic venous ulcer (VU) is a severe manifestation of progressive chronic venous insufficiency of the lower extremities. Characterized by a prolonged disease course, high recurrence rates, and considerable therapeutic challenges, VU markedly impairs patients’ quality of life and imposes a substantial healthcare burden (Simka, 2010; Labropoulos et al., 2012; Bonkemeyer Millan et al., 2019; Iceton et al., 2024; Tan et al., 2024). Although current therapeutic approaches have become increasingly diversified, including compression therapy (Sun et al., 2025), pharmacological interventions (Cerbone et al., 2015), and surgical management (Bolton, 2021), a proportion of patients continue to experience delayed healing or non-healing wounds. This observation suggests that the underlying pathogenesis may involve more complex molecular regulatory networks (Kanta and Zavadakova, 2020; Riaz et al., 2025). Therefore, in-depth identification of key regulatory factors contributing to impaired healing in VU may provide novel targets for precision therapy in chronic wounds.

Nuclear receptor subfamily 4 group A member 3 (NR4A3) participates broadly in diverse physiological and pathological processes (Martinez-Gonzalez et al., 2021), including cell proliferation (Ozsvath and Bush, 2020), migration (Lin et al., 2022), metabolic regulation (Ma et al., 2024), and modulation of immune responses (Odagiu et al., 2020). Recent studies have indicated that NR4A3 plays an important role in wound healing, particularly in regulating keratinocyte function (Sugimoto-Sawada et al., 2025), promoting cell migration (Labropoulos et al., 2012), and suppressing excessive inflammatory responses (Odagiu et al., 2020). Nevertheless, the specific role of NR4A3 in chronic venous ulcers remains unclear. Given its potential regulatory effects in immune and inflammatory processes, we hypothesized that NR4A3 may play a critical role in the non-healing state of VU.

In the present study, bioinformatics approaches were employed using the GSE174661 dataset to identify differentially expressed genes (DEGs) associated with chronic VU, with particular focus on NR4A3 and its related signaling pathways. Kyoto Encyclopedia of Genes and Genomes (KEGG) pathway enrichment analysis and Venn diagram intersection analysis were performed to screen for key pathways and genes potentially involved in chronic wound healing. Furthermore, an NR4A3 knockdown cell model was established, and the PI3K/Akt signaling pathway was activated to validate the potential role of NR4A3 in chronic wounds. This study aims to elucidate the specific function of NR4A3 in chronic venous ulcers and to provide a theoretical basis for the development of novel therapeutic targets and strategies.

## Materials and methods

### Materials

The human immortalized keratinocyte cell line HaCaT (CRL-10482; American Type Culture Collection, ATCC) was used in this study. The Cell Counting Kit-8 (CCK-8) kits (GK10001; GLPBIO, CA, USA) was applied for cell viability assays. Enzyme-linked immunosorbent assay (ELISA) kits for interleukin-8 (IL-8; E-EL-H6008, Elabscience, Wuhan, China), interleukin-6 (IL-6; E-EL-H6156; Elabscience), and C-X-C motif chemokine ligand 5 (CXCL5; E-EL-H0046; Elabscience) were utilized. RNA extraction reagent (P0013C, Beyotime Biotechnology, Shanghai, China), complementary DNA (cDNA) synthesis kit (RK21203, ABclonal, MA, USA), and SYBR Green Fast quantitative polymerase chain reaction (qPCR) Mix (RK21203, ABclonal) were used for gene expression analysis. The bicinchoninic acid (BCA) protein quantification kit (P0012, Beyotime Biotechnology) was used for protein concentration determination. Transwell chambers (3422, Corning, NY, USA), phosphate-buffered saline (PBS; G4200-500ML; Servicebio, Wuhan, China), and enhanced chemiluminescence reagent (MA0186, Beyotime Biotechnology) were also employed.

Primary antibodies included glyceraldehyde-3-phosphate dehydrogenase (GAPDH; 60004-1-Ig, 1:5000; Proteintech, IL, USA), PI3K (4249, 1:1000; Cell Signaling Technology, CST, MA, USA), Akt (9272, 1:1000; CST), phosphorylated PI3K (p-PI3K; 4228, 1:1000; CST), phosphorylated Akt (p-Akt; 4060, 1:1000; CST), GSK3β, p-GSK3β, mTOR and p-mTOR (all 1:1000; CST). Horseradish peroxidase (HRP)-conjugated secondary antibodies included goat anti-rabbit immunoglobulin G (IgG; GB23303, 1:5000; Servicebio) and goat anti-mouse IgG (GB23301, 1:5000; Servicebio).

Equipment included an inverted microscope (Motic, Xiamen, China), microplate reader (Multiskan FC, Thermo Fisher Scientific, United States), real-time fluorescence quantitative polymerase chain (RT-qPCR) reaction system (CG-05, Jinge Scientific Instrument, China), PCR amplifier (ETC821M, Dongsheng Xingye Scientific Instrument, China), electrophoresis power supply (WIX-EP600, WIX, China), electrophoresis tank (WIX-miniPRO4, WIX), protein transfer system (WIX-miniBLOT4, WIX), chemiluminescence imaging system (SH-523, Zhonghua, China), and carbon dioxide (CO_2_) incubator (HF151, Lishen Scientific Instrument, Shanghai, China).

### Bioinformatics analysis

The gene expression dataset GSE174661 was retrieved from the Gene Expression Omnibus (GEO) database, encompassing 20 human skin tissue samples categorized into three groups: normal skin (Skin, n=5), ordinary acute wound (OW, n=10), and chronic venous ulcer (VU, n=5). Patient Characteristics and Sample Selection Criteria: Skin: Samples were obtained from healthy volunteers with no history of chronic wounds, venous insufficiency, dermatological disorders, or systemic inflammatory conditions; age- and sex-matched to wound groups to minimize confounding variables; OW: Acute, non-chronic wounds (e.g., surgical incisions, traumatic wounds) with healing duration < 4 weeks, no clinical signs of infection or venous insufficiency, and confirmed normal peripheral vascular function; VU: Diagnosed according to CEAP classification (C6) with ulcers persisting for ≥ 12 weeks, confirmed venous reflux via Doppler ultrasound, and exclusion of arterial, diabetic, or other non-venous etiologies.

Data preprocessing and differential expression analysis were conducted using R software and the limma package. DEGs were screened using the criteria |log_2_ fold change (FC)| > 1 and *adjusted p* < 0.05. Results were visualized using volcano plots. To further identify significantly altered genes across groups, Venn diagram intersection analysis was performed to determine key genes that were downregulated in Skin versus VU and upregulated in OW versus VU comparisons. KEGG pathway enrichment analysis was performed using the ClusterProfiler package to annotate DEGs and identify major signaling pathways associated with wound repair and inflammatory responses. A *p* value < 0.05 was considered statistically significant for enrichment analysis. Heatmaps based on DEGs were generated to illustrate expression patterns across different groups.

### Cell culture and treatment

HaCaT cells were cultured in DMEM supplemented with 10% FBS, 100 U/mL penicillin, and 100 μg/mL streptomycin to prevent bacterial contamination. HaCaT cells were maintained at 37 °C in a humidified incubator containing 5% CO_2_. When HaCaT cells reached approximately 80% confluence, they were passaged using 0.25% trypsin (P0147; Beyotime Biotechnology), collected by centrifugation, and reseeded at an appropriate density.

When HaCaT cells reached 80% confluence, an NR4A3 knockdown model was established using short hairpin ribonucleic acid (shRNA) technology via liposome-mediated transfection. Cells were divided into the following groups: Control group (Cells were treated with an equal volume of phosphate-buffered saline for 24 hours); Tumor necrosis factor-alpha (TNF-α) stimulation group (Cells were treated with 50 ng/mL tumor necrosis factor-alpha for 24 hours, this group was used to simulate the inflammatory microenvironment of chronic venous ulcers and detect the expression of NR4A3 as well as the phosphorylation levels of GSK3β and mTOR); NR4A3 knockdown group (HaCaT cells were transfected with NR4A3-specific shRNA plasmids and cultured for 24 hours); PI3K/Akt activation group (Cells were treated with the PI3K/Akt agonist 740Y-P [10 μM] for 24 hours); PI3K/Akt inhibition group (Cells were treated with PI3K/Akt inhibitor LY294002 for 24 hours to suppress endogenous PI3K/Akt signaling); and NR4A3 knockdown + PI3K/Akt activation group (Cells were first subjected to NR4A3 knockdown and subsequently treated with 740Y-P [10 μM] for 24 hours to activate the PI3K/Akt signaling pathway for rescue verification).

### Cell viability assay

HaCaT cells were seeded into 96-well plates at a density of 2 × 10^4^ cells per well. After 24 hours of treatment, 10 μL of CCK-8 reagent was added to each well and incubated for an additional 2 hours. Absorbance at 450 nm was measured using a microplate reader, and cell viability was expressed as a percentage. All experiments were performed in triplicate, and data were presented as mean ± standard deviation (x̄ ± SD).

### ELISA

The concentrations of IL-6, IL-8, and CXCL5 in the culture supernatants were measured using corresponding ELISA kits (Elabscience) according to the manufacturer’s instructions. Absorbance was measured using a microplate reader, and concentrations were calculated based on standard curves.

### RT-qPCR

Total RNA was extracted using RNAzol reagent (Beyotime Biotechnology), and RNA concentration and purity were assessed using a NanoDrop spectrophotometer. Complementary DNA was synthesized using the ABclonal reverse transcription kit. Quantitative polymerase chain reaction was performed using SYBR Green Fast qPCR Mix with glyceraldehyde-3-phosphate dehydrogenase as the internal reference gene. The amplification conditions were as follows: pre-denaturation at 95 °C for 3 minutes; 40 cycles of 95 °C for 5 seconds and 58 °C for 30 seconds. Relative gene expression levels were calculated using the 2^−ΔΔCt^ method.

Primer sequences were as follows: GAPDH (forward 5’-TGCACCACCAACTGCTTAGC-3’, reverse 5’-GGCATGGACTGTGGTCATGAG-3’); NR4A3 (forward 5’-CAGGATGAAGACCGCCTATTG-3’, reverse 5’-TGTTCGGTGACGGTAAAAGTC-3’); IL-6 (forward 5’-GAGGATACCACTCCCAACAGACC-3’, reverse 5’-AAGTGCATCATCGTTGTTCATACA-3’); IL-8 (forward 5’-ACTGAGAGTGATTGAGAGTGGAC-3’, reverse 5’-AACCCTCTGCACCCAGTTTTC-3’); CXCL5 (forward 5’-GGTGCTGTGCTCTTGTGC-3’, reverse 5’-TGAGCAACACACTTGGATGA-3’).

### Western blotting (WB)

Total cellular protein was extracted using radioimmunoprecipitation assay (RIPA) buffer, and protein concentration was determined using the bicinchoninic acid assay. Equal amounts of protein were separated by 10% sodium dodecyl sulfate–polyacrylamide gel electrophoresis (SDS-PAGE) and transferred onto polyvinylidene fluoride membranes. Membranes were blocked with 5% non-fat milk and incubated overnight at 4 °C with primary antibodies against NR4A3, PI3K, Akt, p-PI3K, p-Akt, GSK3β, p-GSK3β, mTOR and p-mTOR. After incubation with HRP-conjugated secondary antibodies, protein bands were visualized using a chemiluminescence imaging system. Densitometric analysis of band gray values was performed using ImageJ software. For phosphorylated proteins, the relative phosphorylation level was calculated as the ratio of phosphorylated protein gray value to the corresponding total protein gray value. All total protein bands were further normalized to the internal reference GAPDH gray value to eliminate unequal sample loading differences.

### Transwell migration and invasion

Following treatment, HaCaT cells were resuspended in serum-free medium at a density of 2–5 × 10^5^ cells/mL. Two hundred microliters of cell suspension were added to the apical chamber of the Transwell insert, while 600 μL of medium containing 10% FBS was added to the basolateral chamber as a chemoattractant. For migration assays, cells were directly incubated. For invasion assays, the Transwell membrane was pre-coated with Matrigel matrix and incubated at 37 °C for 30 minutes before cell seeding. After 24 hours of incubation at 37 °C with 5% CO_2_, cells were gently washed with PBS, fixed with 4% paraformaldehyde for 15 minutes, and stained with 0.1% crystal violet. Cells were counted under an inverted microscope, and quantitative analysis was performed using ImageJ software.

### Wound healing assay

HaCaT cells were seeded into 6-well plates at a density of 5 × 10^5^ cells per well. When approximately 80% confluence was reached, a linear scratch was created in the center of each well using a sterile 200 μL pipette tip. Images were captured at 0 and 24 hours, and the percentage of wound closure was calculated.

### Statistical analysis

Data were expressed as x̄ ± SD. Comparisons between two groups were performed using the independent-samples *t*-test, while comparisons among multiple groups were conducted using one-way analysis of variance (ANOVA). A *p* value < 0.05 was considered statistically significant. All statistical analyses were performed using Statistical Package for the Social Sciences (SPSS) version 26.0 software.

## Results

### Identification of NR4A3 as a DEG in chronic wounds and pathway enrichment analysis

**Figure 1:**
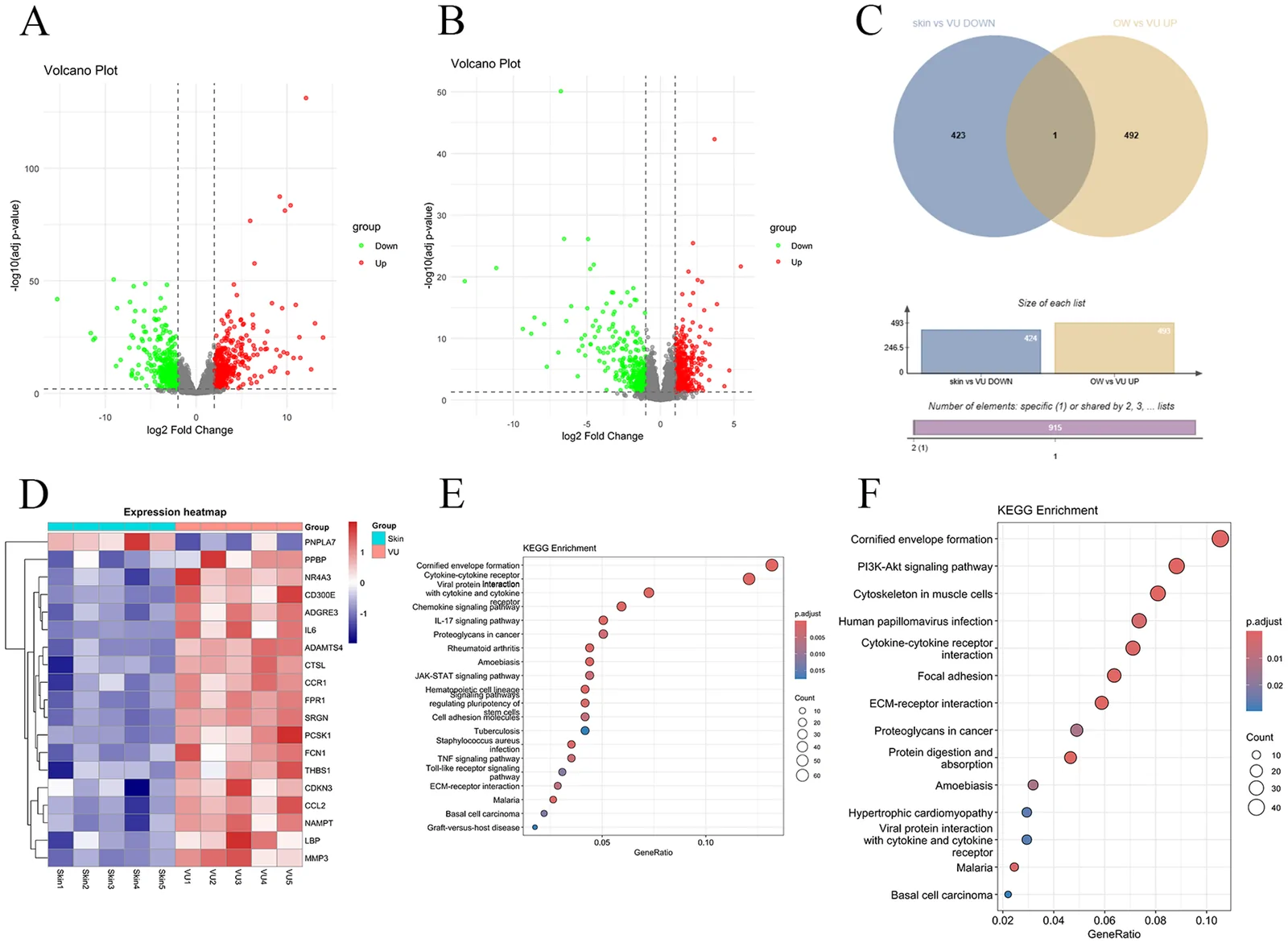
Differential expression analysis and functional enrichment results based on the GSE174661 dataset in chronic VU tissues. **(A, B)** Volcano plots showing DEGs in VU tissues compared to Skin **(A)** and OW **(B, C)** Venn diagram illustrating the intersection of DEGs from the Skin vs. VU and OW vs. VU comparisons. **(D)** Heatmap depicting the expression levels of the intersecting gene, NR4A3, in Skin, OW, and VU samples. **(E, F)** KEGG pathway enrichment analysis of DEGs identified in the Skin vs. VU **(E)** and OW vs. VU **(F)** comparisons.

Differential expression analysis was performed using the GSE174661 dataset from the NCBI GEO database, which comprises RNA sequencing data from human skin tissue samples. This dataset, generated on the Illumina HiSeq 2500 platform, includes three distinct sample groups with clear annotations: normal skin (Skin), ordinary wounds (OW), and chronic VU. Comparing Skin vs. VU identified 837 DEGs, with 412 upregulated and 425 downregulated. The comparison of OW vs. VU yielded 857 DEGs, consisting of 492 upregulated and 365 downregulated genes. The distribution of these DEGs is presented in volcano plots (**Figures 1A, B**).

By intersecting the DEGs from both comparisons, genes with opposing expression trends were identified. A single gene, the transcription factor NR4A3, was found to be significantly downregulated in Skin vs. VU and concurrently upregulated in OW vs. VU (**Figure 1C**). Its expression was markedly lower in VU tissues compared to both Skin and OW groups. This expression pattern was further validated by heatmap visualization (**Figure 1D**).

KEGG pathway enrichment analysis of the identified DEGs revealed significant enrichment in several signaling networks critical for cell migration, differentiation, and inflammatory regulation, including the PI3K-Akt signaling pathway, ECM-receptor interaction, and cytokine-cytokine receptor interaction (**Figures 1E, F**). Notably, the PI3K/Akt pathway was consistently enriched among DEGs from both comparisons.

### NR4A3 expression following TNF-α stimulation and gene knockdown

**Figure 2:**
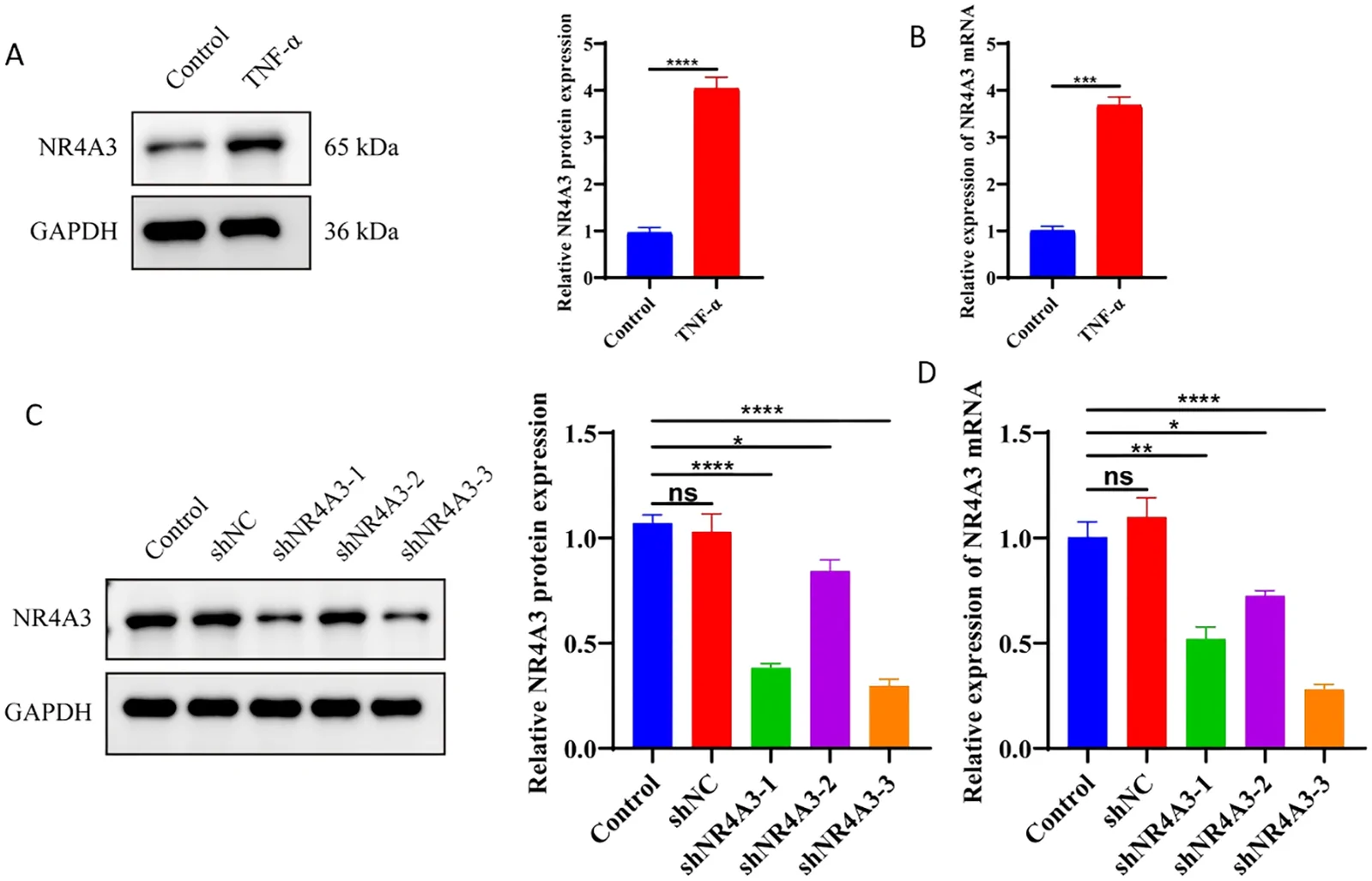
NR4A3 expression following TNF-α stimulation and validation of RNA interference efficiency. **(A)** Protein levels of NR4A3 in HaCaT cells after TNF-α stimulation, assessed by WB. **(B)** Relative mRNA expression levels of NR4A3 after TNF-α stimulation, determined by RT-qPCR. **(C)** Protein expression levels of NR4A3 in HaCaT cells following transfection with different shRNAs (shNR4A3-1, shNR4A3-2, shNR4A3-3) compared with the Control and shNC groups. **(D)** Relative mRNA expression levels of NR4A3 after transfection with the indicated shRNAs. *p < 0.05, **p < 0.01, ***p < 0.001, ****p < 0.0001; ns indicates no significant difference All western blot experiments were independently repeated three times; densitometric quantification is shown as mean ± SD from three biological replicates.

To explore the role of NR4A3 in chronic VU, we assessed the effect of TNF-α on its expression. Treatment with TNF-α significantly increased both protein and mRNA levels of NR4A3 (**Figures 2A, B**). To further investigate NR4A3 function, we knocked down the gene using shRNA technology. A significant reduction in NR4A3 protein and mRNA levels was observed in the shNR4A3 group compared to controls (**Figures 2C, D**), which showed successful gene knockdown.

### Effects of NR4A3 knockdown on cell proliferation, migration, invasion, and inflammatory responses

**Figure 3:**
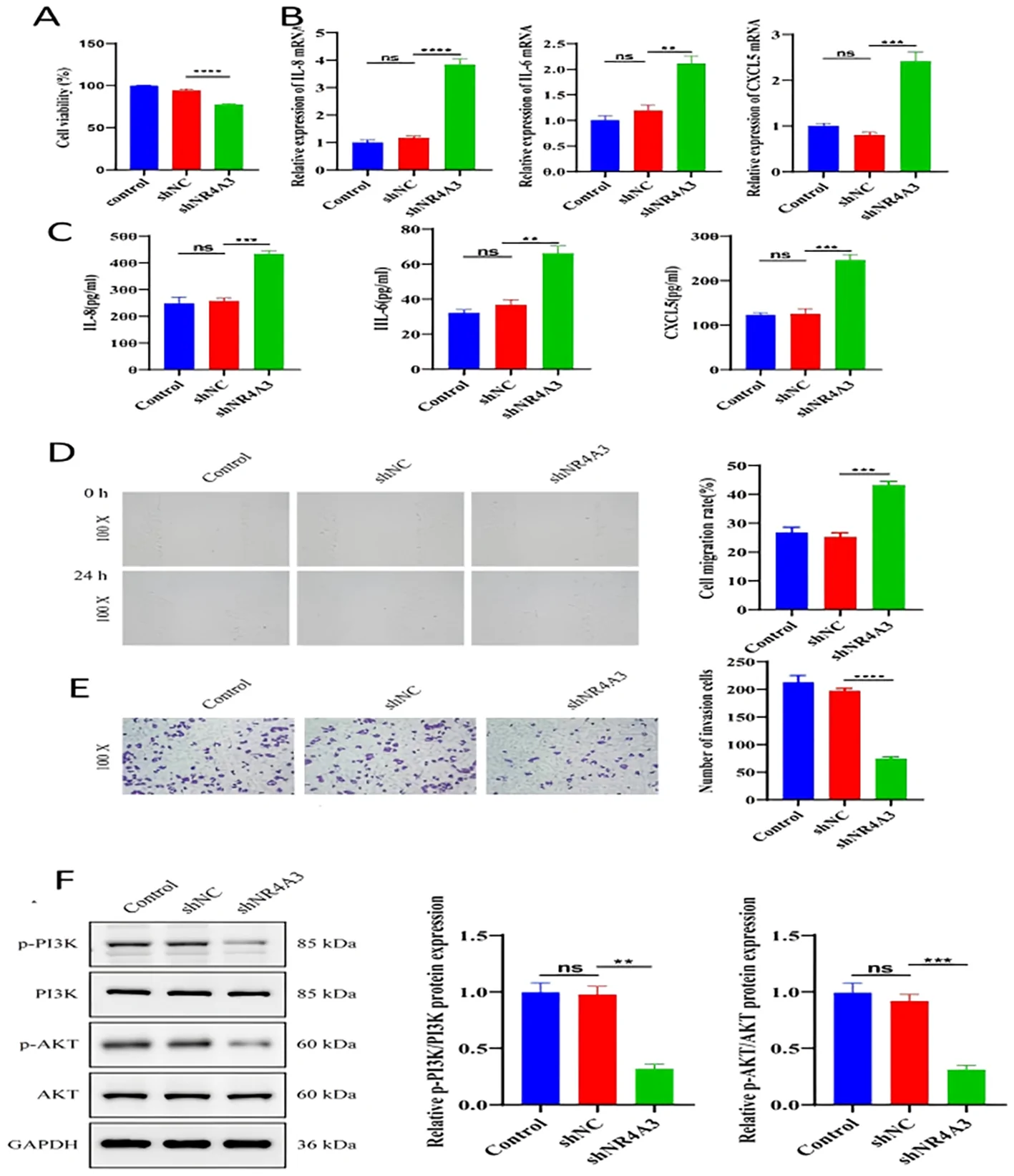
Effects of NR4A3 knockdown on inflammatory cytokines, as well as migration and invasion in HaCaT cells. **(A)** Cell viability in the Control, shNC, and shNR4A3 groups, assessed by the CCK-8 assay. **(B)** Relative mRNA levels of IL-6, IL-8, and CXCL5 in the Control, shNC, and shNR4A3 groups, measured by RT-qPCR. **(C)** Protein concentrations of IL-6, IL-8, and CXCL5 in the cell culture supernatant of the Control, shNC, and shNR4A3 groups, measured by ELISA. **(D)** Representative images (100× magnification) and quantitative analysis of cell migration ability in the Control, shNC, and shNR4A3 groups cells, assessed by wound healing assay. **(E)** Representative images (100× magnification) and quantitative analysis of cell invasion ability in the Control, shNC, and shNR4A3 groups cells, assessed by Transwell assay. **(F)** WB analysis and quantification of total and phosphorylated protein levels of key components of the PI3K/Akt signaling pathway (PI3K, p-PI3K, Akt, p-Akt) in the Control, shNC, and shNR4A3 groups. *p < 0.05, **p < 0.01, ***p < 0.001, ****p < 0.0001; ns indicates no significant difference. All western blot experiments were independently repeated three times; densitometric quantification is shown as mean ± SD from three biological replicates.

The effect of NR4A3 on keratinocyte function was examined. Cell proliferation, assessed by the CCK-8 assay, was significantly inhibited following NR4A3 knockdown, with the shNR4A3 group showing a lower proliferation rate compared to the control group (**Figure 3A**). Furthermore, the role of NR4A3 in modulating inflammatory factors was evaluated. RT-qPCR and ELISA assays demonstrated that NR4A3 knockdown significantly upregulated both the mRNA and protein expression of IL-8, IL-6, and CXCL5 (**Figures 3B, C**). Regarding cellular functions, wound healing assay results indicated that NR4A3 knockdown promoted cell migration (**Figure 3D**). Conversely, Transwell invasion assays showed that NR4A3 knockdown suppressed the invasive capacity of cells (**Figure 3E**). Additionally, the phosphorylation levels of the PI3K/Akt signaling pathway were significantly decreased upon NR4A3 knockdown (**Figure 3F**).

### Restoration of cellular function following NR4A3 gene knockout using PI3K/Akt agonists and inhibitors

**Figure 4:**
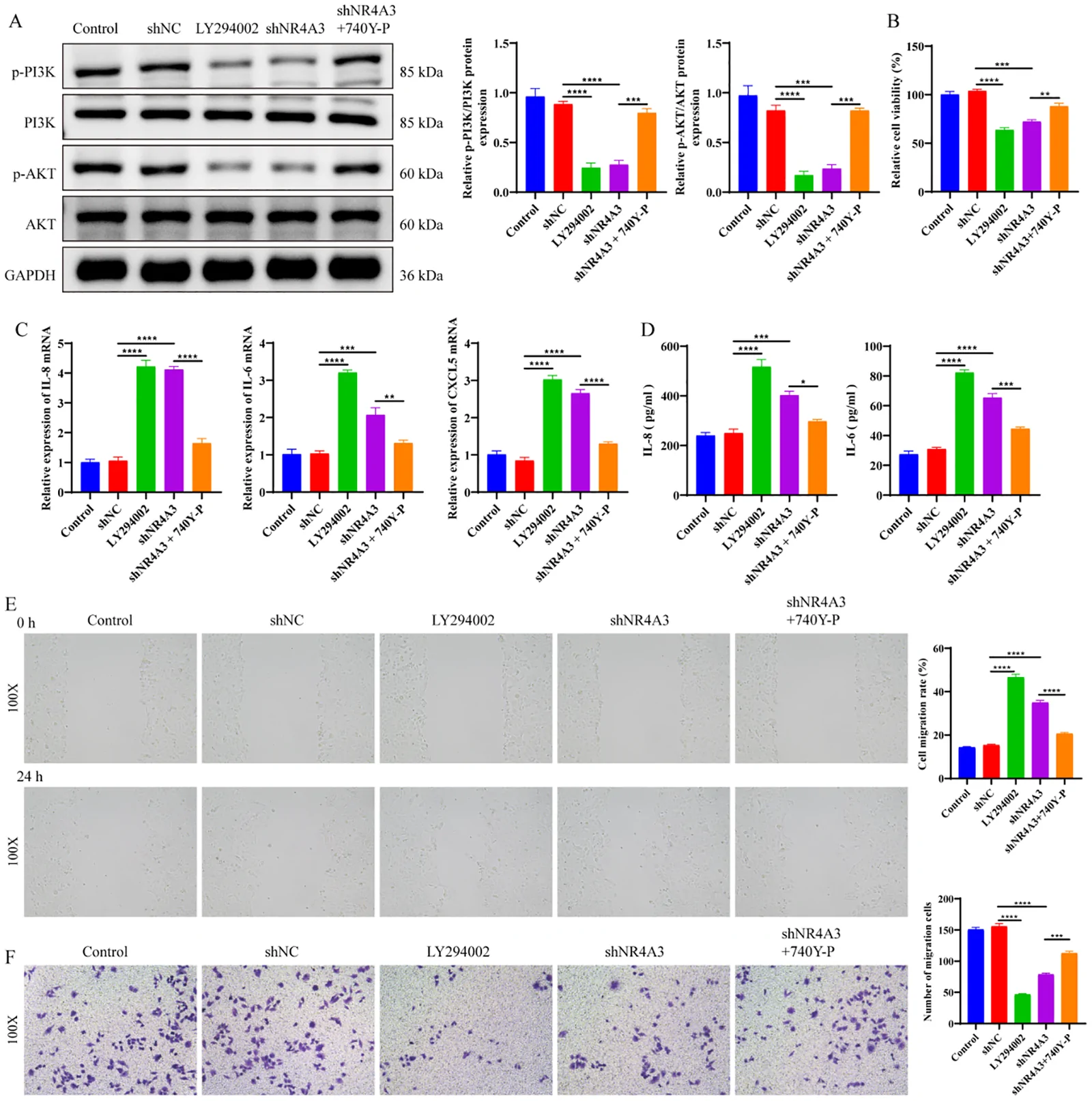
NR4A3-associated changes in pro-inflammatory responses and migration in keratinocytes may involve the PI3K/Akt pathway. **(A)** WB analysis and quantification of total and phosphorylated levels of key components of the PI3K/Akt signaling pathway (PI3K, p-PI3K, Akt, p-Akt) in the indicated groups. **(B)** Cell viability in the indicated groups, assessed by the CCK-8 assay. **(C)** Relative mRNA expression levels of IL-6, IL-8, and CXCL5 in the indicated groups, measured by RT-qPCR. **(D)** Protein concentrations of IL-6, IL-8, and CXCL5 in the cell culture supernatant of the indicated groups, measured by ELISA. **(E)** Representative images (100× magnification) and quantitative analysis of cell migration ability in the indicated groups, assessed by wound healing assay. **(F)** Representative images (100× magnification) and quantitative analysis of cell invasion ability in the indicated groups, assessed by Transwell assay. *p < 0.05, **p < 0.01, ***p < 0.001, ****p < 0.0001; ns indicates no significant difference. All western blot experiments were independently repeated three times; densitometric quantification is shown as mean ± SD from three biological replicates.

We treated cells with the PI3K/Akt agonist 740Y-P and inhibitor LY294002. Relative to the shNC group, phosphorylation of PI3K and Akt was notably decreased in the shNR4A3 and LY294002 groups, whereas this trend was reversed in the shNR4A3 + 740Y-P group (**Figure 4A**). CCK-8 assays showed suppressed cell proliferation in the shNR4A3 and LY294002 groups, while 740Y-P treatment restored the proliferative ability of shNR4A3 cells (**Figure 4B**). qPCR results revealed upregulated mRNA levels of pro-inflammatory factors (IL-6, IL-8 and CXCL5) in shNR4A3 and LY294002 groups, and such upregulation was abrogated by 740Y-P co-treatment (**Figure 4C**). Consistently, ELISA analysis confirmed higher secretion of IL-6, IL-8 and CXCL5 in the supernatant of shNR4A3 and LY294002 cells, which was also downregulated upon 740Y-P intervention (**Figure 4D**). Wound scratch assays showed elevated cell migration in shNR4A3 and LY294002 groups (**Figure 4E**), while Transwell invasion capacity was impaired in these two groups (**Figure 4F**). Moreover, 740Y-P supplementation weakened cell migration and rescued the invasive potential of shNR4A3-silenced cells.

### The effect of NR4A3 overexpression on the proliferation, migration, invasion, inflammatory response, and AKT/PI3K pathway in HaCaT cells

**Figure 5:**
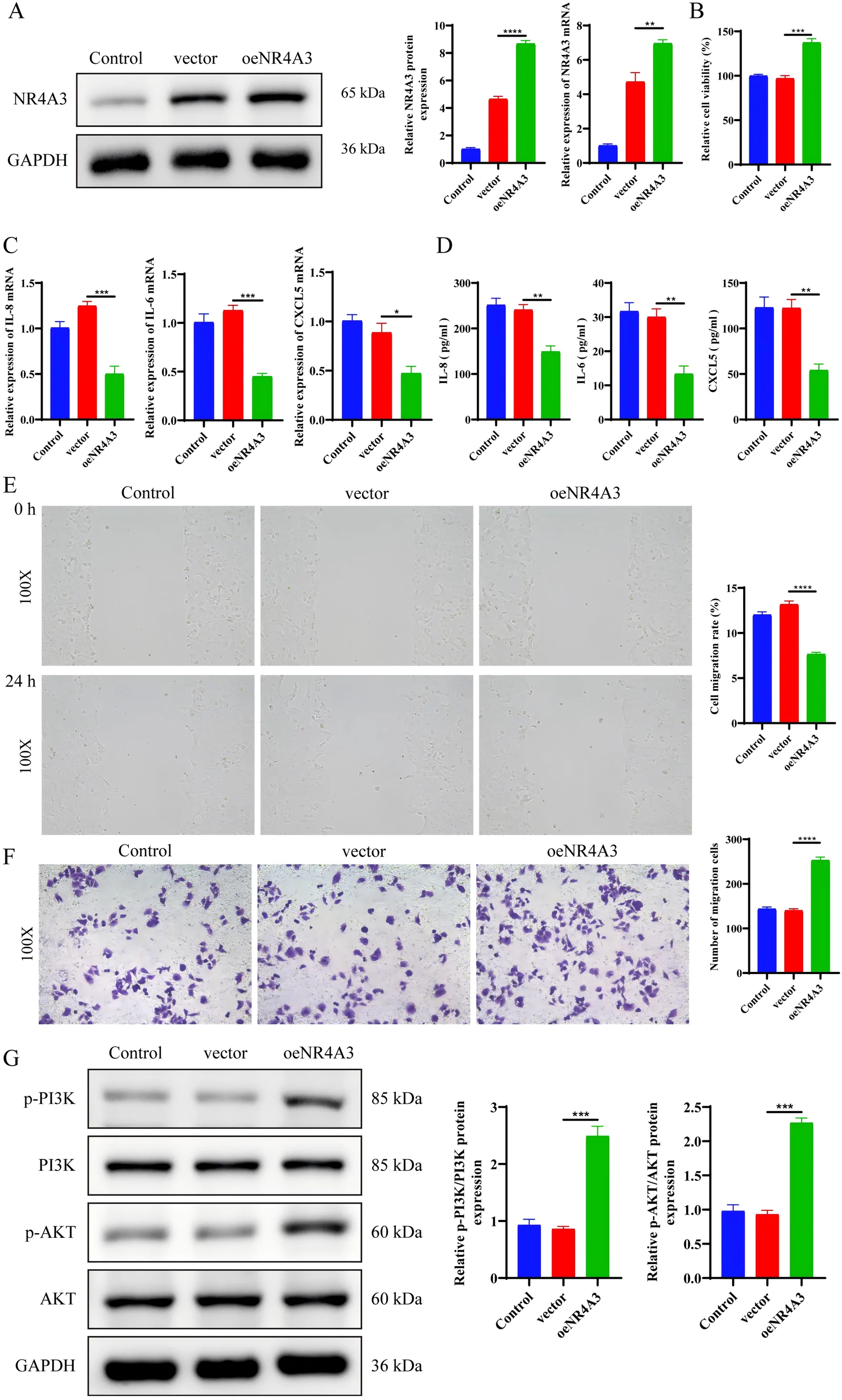
The effect of NR4A3 overexpression on the proliferation, migration, invasion, inflammatory response, and PI3K/Akt pathway in HaCaT cells. **(A)** Protein and mRNA expression levels of NR4A3 in the Control, empty-vector, and oeNR4A3 groups, assessed by Western blotting and RT-qPCR, respectively. detected by Western blot and RT-qPCR. **(B)** Cell viability in the indicated groups, assessed by the CCK-8 assay. **(C)** Relative mRNA expression levels of IL-6, IL-8, and CXCL5 in the indicated groups, measured by RT-qPCR. **(D)** Protein concentrations of IL-6, IL-8, and CXCL5 in the cell culture supernatant of the indicated groups, measured by ELISA. **(E)** Representative images (100× magnification) and quantitative analysis of cell migration ability in the indicated groups, assessed by wound healing assay. **(F)** Representative images (100× magnification) and quantitative analysis of cell invasion ability in the indicated groups, assessed by Transwell assay. **(G)** WB analysis and quantification of total and phosphorylated levels of key components of the PI3K/Akt signaling pathway (PI3K, p-PI3K, Akt, p-Akt) in the indicated groups. *p < 0.05, **p < 0.01, ***p < 0.001, ****p < 0.0001; ns indicates no significant difference. All western blot experiments were independently repeated three times; densitometric quantification is shown as mean ± SD from three biological replicates.

NR4A3 overexpression markedly increased its mRNA and protein levels in the oeNR4A3 group relative to the vector group (**Figure 5A**). CCK-8 assays showed that NR4A3 overexpression significantly promoted cell proliferation (**Figure 5B**). qPCR results indicated decreased mRNA levels of pro-inflammatory factors IL-6, IL-8 and CXCL5 in oeNR4A3 cells (**Figure 5C**), and ELISA verified the corresponding reduction in the secretion of these cytokines in cell supernatants (**Figure 5D**). Functional experiments demonstrated suppressed cell migration and strengthened invasive capacity in the oeNR4A3 group (**Figure 5E, F**). Furthermore, Western blot analysis confirmed higher phosphorylation of PI3K and Akt in NR4A3-overexpressing cells (**Figure 5G**).

### TNF-α stimulation increases the phosphorylation of GSK3β and mTOR in HaCaT keratinocytes

**Figure 6:**
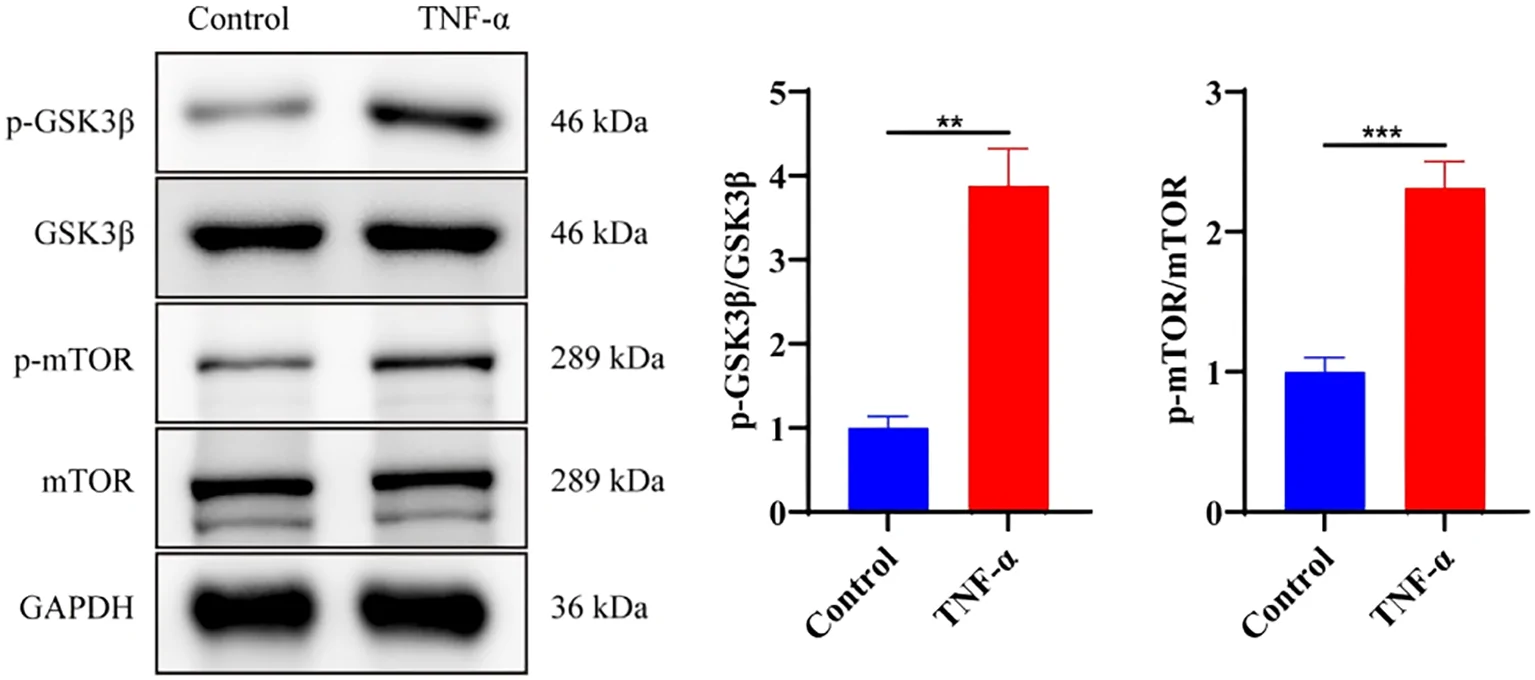
TNF-α stimulation increases the phosphorylation of GSK3β and mTOR in HaCaT keratinocytes. Western blot bands of phosphorylated and total GSK3β, phosphorylated and total mTOR in the Control group and TNF-α treatment group. GAPDH was applied as the internal loading control for western blot detection. *p < 0.05, **p < 0.01, ***p < 0.001, ****p < 0.0001; ns indicates no significant difference. All western blot experiments were independently repeated three times; densitometric quantification is shown as mean ± SD from three biological replicates.

Western blot analysis revealed that TNF-α stimulation markedly elevated the protein expression levels of p-GSK3β and p-mTOR compared with the Control group (**Figure 6A**).

### NR4A3 regulates the phosphorylation level of the PI3K/Akt signaling pathway in keratinocytes

**Figure 7:**
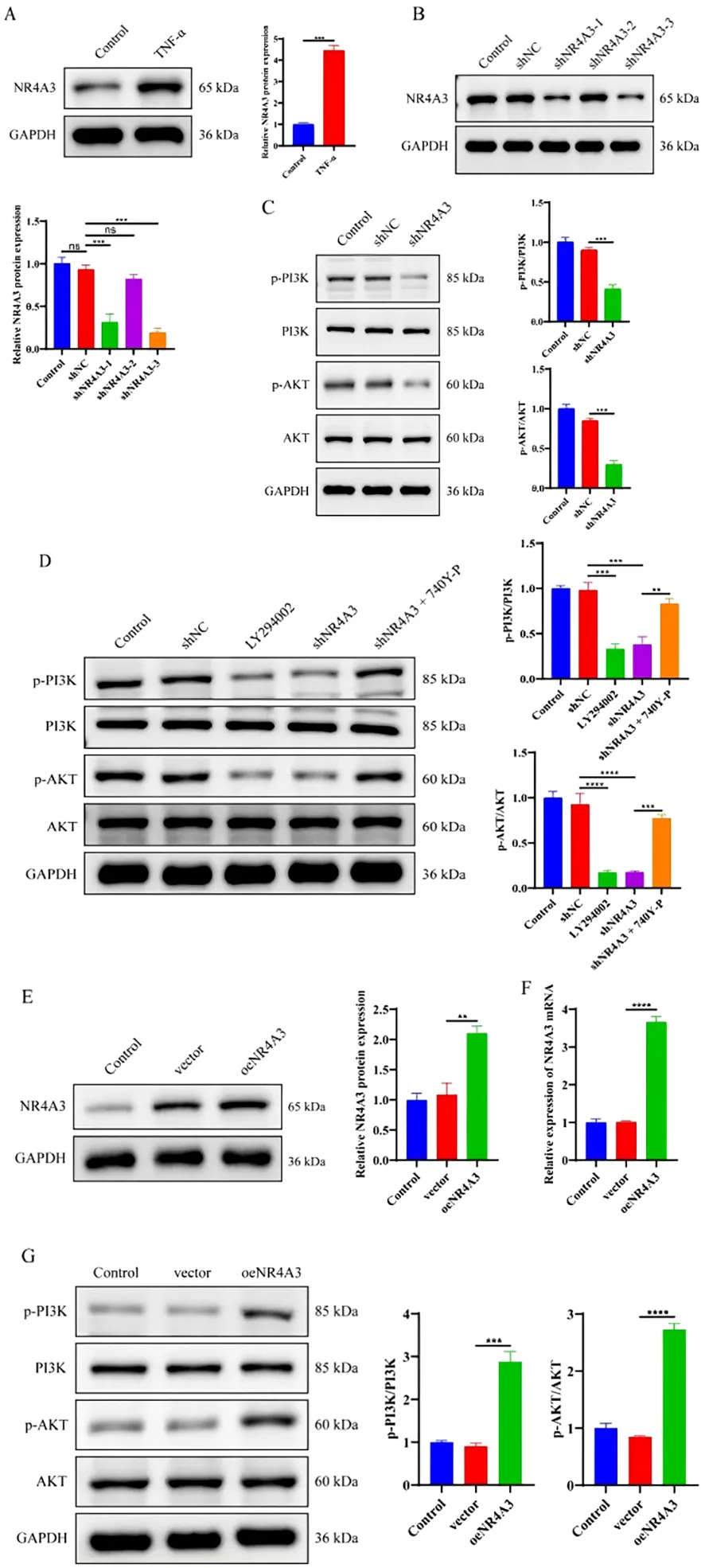
NR4A3-associated changes in PI3K/Akt phosphorylation in HaCaT keratinocytes. **(A)** Western blot analysis of NR4A3 protein abundance in control and TNF-α-stimulated HaCaT cells. **(B)** Immunoblot validation of knockdown efficiency for three independent NR4A3 shRNA constructs (shNR4A3-1, shNR4A3-2, shNR4A3-3) versus blank control and shNC groups; shNR4A3–3 exerted the strongest silencing effect. **(C)** Loss of NR4A3 decreased the phosphorylation of PI3K and Akt in HaCaT cells. **(D)** Rescue assay: PI3K/Akt agonist 740Y-P recovered the declined p-PI3K/p-Akt levels triggered by NR4A3 knockdown, whereas PI3K/Akt inhibitor LY294002 aggravated pathway inhibition. **(E, F)** Western blot and RT-qPCR confirmed successful overexpression of NR4A3 in oeNR4A3 group compared with empty vector control. **(G)** Ectopic overexpression of NR4A3 was accompanied by increased PI3K and Akt phosphorylation. GAPDH was used as the internal loading control for all western blot experiments. *p < 0.05, **p < 0.01, ***p < 0.001, ****p < 0.0001; ns indicates no significant difference. All western blot experiments were independently repeated three times; densitometric quantification is shown as mean ± SD from three biological replicates.

Compared with the Control group, the protein expression level of NR4A3 was significantly upregulated in the TNF-α treatment group (**Figure 7A**). In the NR4A3 gene knockdown experiment, WB results revealed that the protein expression of NR4A3 was decreased in the shNR4A3-1, shNR4A3–2 and shNR4A3–3 groups relative to the Control and NC groups, with the shNR4A3–3 group exhibiting the most pronounced knockdown efficiency (**Figure 7B**). Knockdown of NR4A3 markedly suppressed the phosphorylation levels of PI3K and AKT (**Figure 7C**). Compared with the shNC group, both the shNR4A3 group and LY294002 group displayed remarkable reductions in the protein phosphorylation levels of p-PI3K and p-AKT; the shNR4A3 + 740Y-P group effectively reversed this inhibitory effect and substantially restored the phosphorylation levels of p-PI3K and p-AKT (**Figure 7D**). Western blot and qPCR detection results demonstrated that the oeNR4A3 group presented significantly elevated NR4A3 expression at both mRNA and protein levels when compared with the Vector group (**Figures 7E, F**). Further WB analysis illustrated that overexpression of NR4A3 was accompanied by increased phosphorylation levels of p-PI3K and p-AKT, supporting a functional association between NR4A3 and PI3K/AKT pathway activity (**Figure 7G**).

## Discussion

Chronic VU represents a common and refractory chronic wound condition, in which patients often experience prolonged suffering due to delayed wound healing (Crawford et al., 2017). Although therapeutic approaches have advanced in recent years, the molecular mechanisms underlying impaired healing in chronic wounds remain incompletely understood. In the present study, bioinformatics analysis combined with *in vitro* functional experiments was employed to investigate the expression profile of the transcription factor NR4A3 in VU and its regulatory role in keratinocyte function.

Differential expression analysis revealed that NR4A3 expression was significantly lower in VU tissues compared to Skin and OW tissues. This finding suggests that reduced NR4A3 expression may be associated with the pathological state of chronic wounds. Previous studies have reported that NR4A3 promotes wound healing by regulating cell migration (Weibel, 2017), immune responses (Hiwa et al., 2022), and extracellular matrix remodeling (Zheng et al., 2024). It is hypothesized that decreased NR4A3 expression may lead to impaired cellular function, thereby contributing to delayed healing in chronic wounds (Chen et al., 2023). The observed downregulation of NR4A3 in VU tissue samples aligns with these functional reports, providing clinically relevant clue for further investigation into its role in chronic wounds.

Subsequently, the potential mechanism of NR4A3 in chronic VU was further explored. KEGG pathway enrichment analysis showed that DEGs were significantly enriched in the PI3K/Akt signaling pathway. Our WB results further showed that phosphorylation levels of PI3K and Akt were decreased following NR4A3 knockdown, whereas activation of this pathway using the agonist 740Y-P partially restored cell proliferation and migration. These findings suggest that the PI3K/Akt pathway may mediate the regulatory effects of NR4A3 on keratinocyte function. The PI3K/Akt pathway plays a critical role in cell migration (Huang et al., 2025), proliferation (Liu et al., 2025), and survival (Cao et al., 2023), and accumulating evidence has demonstrated that activation of PI3K/Akt promotes wound healing (Wang et al., 2021; Sun et al., 2022; Zhang et al., 2025). The present experimental results are consistent with this hypothesis and provide preliminary evidence supporting the involvement of this pathway in NR4A3-mediated regulation.

Persistent activation of inflammatory responses is considered a major contributor to delayed wound healing in chronic VU (Raffetto et al., 2020; Fernandez-Guarino et al., 2023). Notably, our scratch wound and Transwell invasion assays exhibited seemingly contradictory phenotypes upon NR4A3 silencing: keratinocyte migratory capacity was enhanced, while invasive potential was markedly suppressed. This divergent cellular behavior can be attributed to distinct regulatory programs governing cytoskeletal remodeling and extracellular matrix (ECM) degradation, which are differentially controlled by the PI3K/Akt cascade. Cell migration mainly relies on dynamic rearrangement of actin cytoskeleton to drive directional planar movement on intact epidermal matrix, whereas cell invasion requires additional secretion of matrix metalloproteinases to degrade dense Matrigel/ECM barriers for deep tissue penetration. Reduced PI3K/Akt activity after NR4A3 knockdown impairs the transcriptional activation of ECM-degrading proteases, resulting in weakened invasive capacity; meanwhile, the sustained inflammatory microenvironment triggered by accumulated IL-6, IL-8 and CXCL5 may involve compensatory cytoskeletal regulatory mechanisms that are not fully dependent on PI3K/Akt signaling, thereby accelerating planar cell migration in scratch wounds. Related studies have indicated that NR4A3 promotes wound healing by modulating immune responses, and its deficiency may lead to excessive inflammatory activation (Crean and Murphy, 2021). The increased inflammatory cytokine levels observed following NR4A3 knockdown in the present study suggest that NR4A3 may participate in wound healing through regulation of local inflammatory responses (Xiao et al., 2020).

Further experiments demonstrated an association between NR4A3 downregulation and extracellular matrix deposition as well as myofibroblast activation. In chronic wounds, extracellular matrix remodeling (Song et al., 2023) and myofibroblast activation (Schuster et al., 2023) represent critical steps in the healing process. Previous studies have shown that NR4A3 promotes extracellular matrix remodeling by regulating fibroblast function (Andreas et al., 2009; Yi et al., 2025). The present data further support this notion, suggesting that NR4A3 deficiency may lead to aberrant extracellular matrix deposition, thereby exacerbating healing impairment in chronic VU.

**Figure 8:**
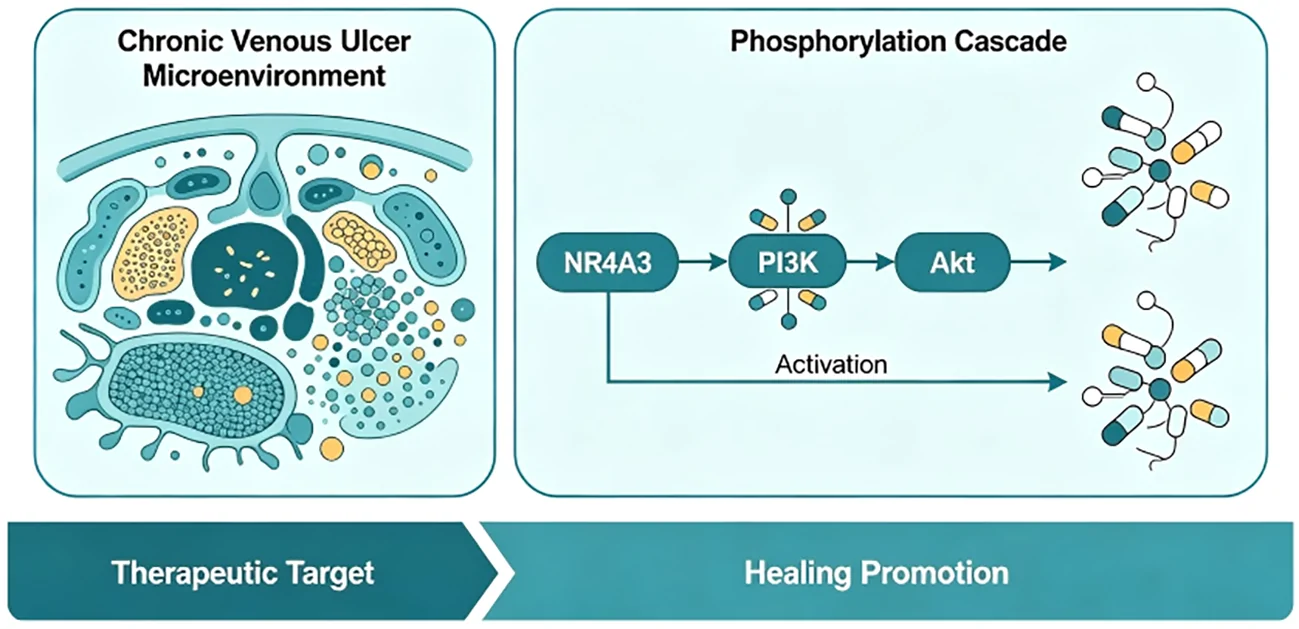
The regulatory mechanism of NR4A3–PI3K/Akt in the healing process of chronic venous ulcers.

In addition to the PI3K/Akt pathway, RNA-seq analysis revealed that differentially expressed genes were not only significantly enriched in the PI3K/Akt signaling pathway but also showed an upward trend in gene sets associated with NF-κB and MAPK pathways in the GSEA enrichment profile. Concurrently, the NF-κB pathway (Lin et al., 2025) and MAPK pathway (Hsu et al., 2024) have been reported to play important roles in chronic wound healing. The present study suggests that NR4A3 may functionally modulate PI3K/Akt pathway activity during chronic VU healing. NR4A3 may be associated with changes in the phosphorylation levels of PI3K and Akt, although direct transcriptional or protein-interaction evidence remains to be established. Downregulation of NR4A3 in VU tissues may weaken PI3K/Akt pathway activity, which in turn could impair keratinocyte proliferation and invasion, trigger abnormal migration and sustained inflammatory responses, and ultimately contribute to delayed wound healing. Activation of the PI3K/Akt pathway using agonist 740Y-P can compensate for the functional defects caused by NR4A3 deficiency, further supporting that NR4A3-associated regulation of keratinocyte biological behaviors and inflammatory responses at least partly involves the PI3K/Akt signaling cascade (**Figure 8**).

Although this study explored the role of NR4A3 in chronic venous ulcer healing via PI3K/Akt signaling through bioinformatics and HaCaT cell assays, several limitations remain. First, mechanistic evidence is insufficient to confirm direct NR4A3 regulation of PI3K/Akt components. We only observed functional correlation and pharmacological rescue without ChIP-qPCR, luciferase reporter or co-IP assays to verify transcriptional or physical interactions. Moreover, TNF-α, 740Y-P and LY294002 were used at single fixed concentrations with no dose- or time-gradient tests, failing to reveal dose/time-dependent effects. Small-molecule modulators also carry potential off-target effects. Future work will use gene silencing, gradient treatments and molecular binding assays to clarify their regulatory relationship. Second, all experiments were performed solely in immortalized HaCaT keratinocytes. This cell line differs from primary patient-derived keratinocytes (Moran et al., 2021), and the monoculture model lacks fibroblasts, endothelial cells and immune cells that constitute the ulcer microenvironment. Limited clinical tissue resources prevented validation in primary cells or co-culture systems in this revision, which will be addressed in follow-up animal and primary cell experiments. Third, Western blot data had insufficient transparency in the initial version, including missing standardized quantification rules, inconsistent loading controls and incompletely presented raw blots. Though we supplemented unified normalization criteria and full uncropped blots in revision, repeated gradient exposure tests and broader detection of PI3K/Akt downstream targets are still needed. Fourth, NR4A3 knockdown induced elevated scratch migration but reduced Transwell invasion. We attributed this to distinct PI3K/Akt control of cytoskeletal rearrangement and ECM degradation, yet we did not detect MMP or actin markers to supply direct molecular proof for this divergent phenotype. Lastly, transcriptomic enrichment implied involvement of NF-κB and MAPK pathways, but this study only focused on PI3K/Akt, leaving crosstalk between multiple inflammatory axes uncharacterized.

Despite these limitations, this study offers a potential direction for the treatment of chronic VU. Patients with chronic VU often face prolonged treatment duration and high recurrence rates, and previous therapeutic strategies are predominantly symptomatic, lacking targeted approaches with well-defined mechanisms (Lazarus et al., 2014; Attaran et al., 2025). The findings of this study suggest that targeting NR4A3 or its downstream PI3K/Akt pathway may represent a potential therapeutic option for chronic VU. Future development of NR4A3-related small molecule agonists or local delivery systems may be considered, although their clinical feasibility and efficacy require further validation through animal models and clinical trials.

In conclusion, *in vitro* evidence suggests that NR4A3 is associated with keratinocyte biological behaviors and inflammatory responses via modulation of the PI3K/Akt signaling pathway, thereby potentially affecting the progression and healing of chronic VU. Reduced NR4A3 expression may impair wound healing through sustained inflammation and abnormal cell migration, while inflammatory TNF-α exposure triggers activation of GSK3β/mTOR alongside compensatory NR4A3 elevation.

## Data Availability

The transcriptomic dataset analyzed in this study is publicly available in the Gene Expression Omnibus (GEO) database under accession number GSE174661. Additional experimental data generated during this study are available from the corresponding author upon reasonable request.
