# Supplementary material for: Impact of NR4A3 on wound healing in chronic venous ulcers and its association with the PI3K/Akt signaling pathway
Source: Front Physiol. 2026 Aug 31;17:1822383. doi: 10.3389/fphys.2026.1822383 (PMC13573241; doi:10.3389/fphys.2026.1822383)

Fig 2-A

1 **2** **3**

NR4A3
65 kDa

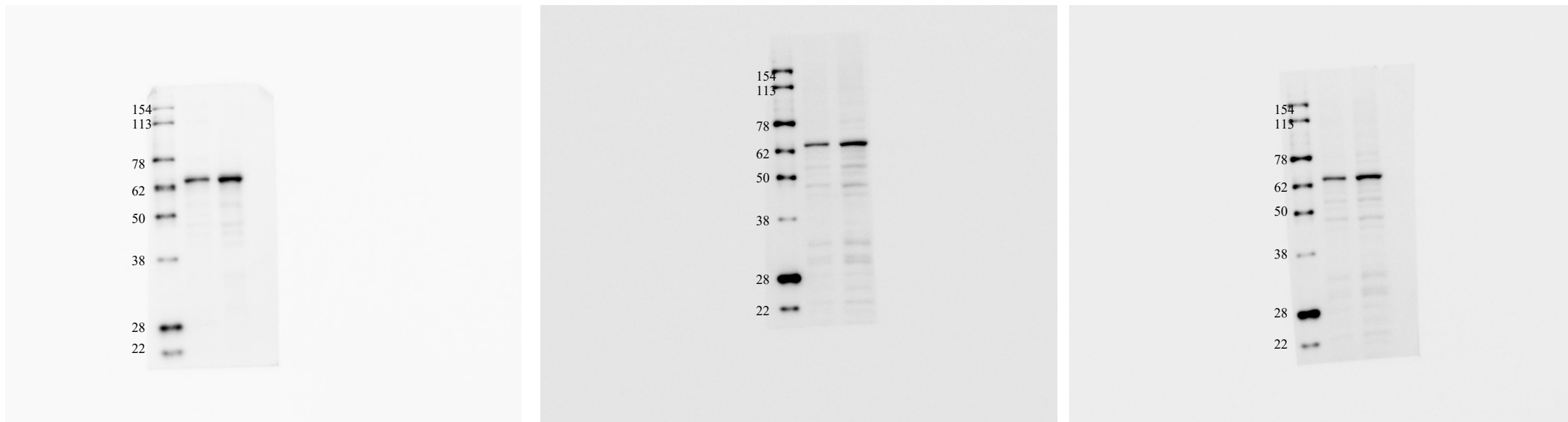

GAPDH
36 kDa

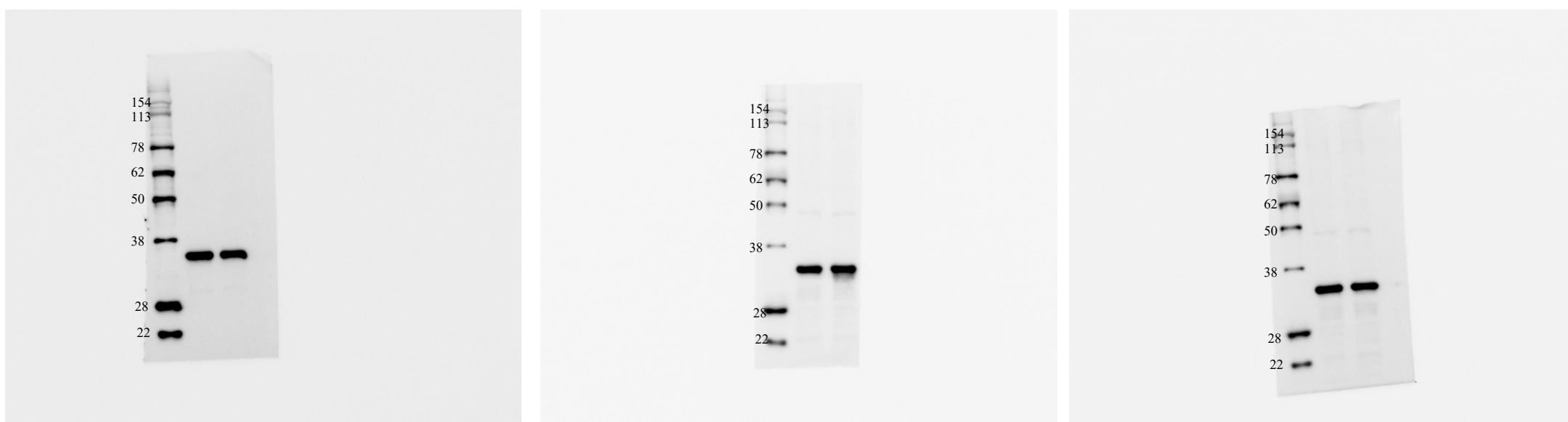

Marker: G2086 Servicebio

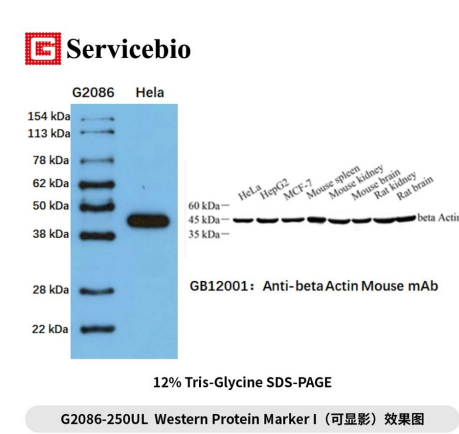

Fig 2-C

	1	2	3

NR4A3
65 kDa

GAPDH
36 kDa

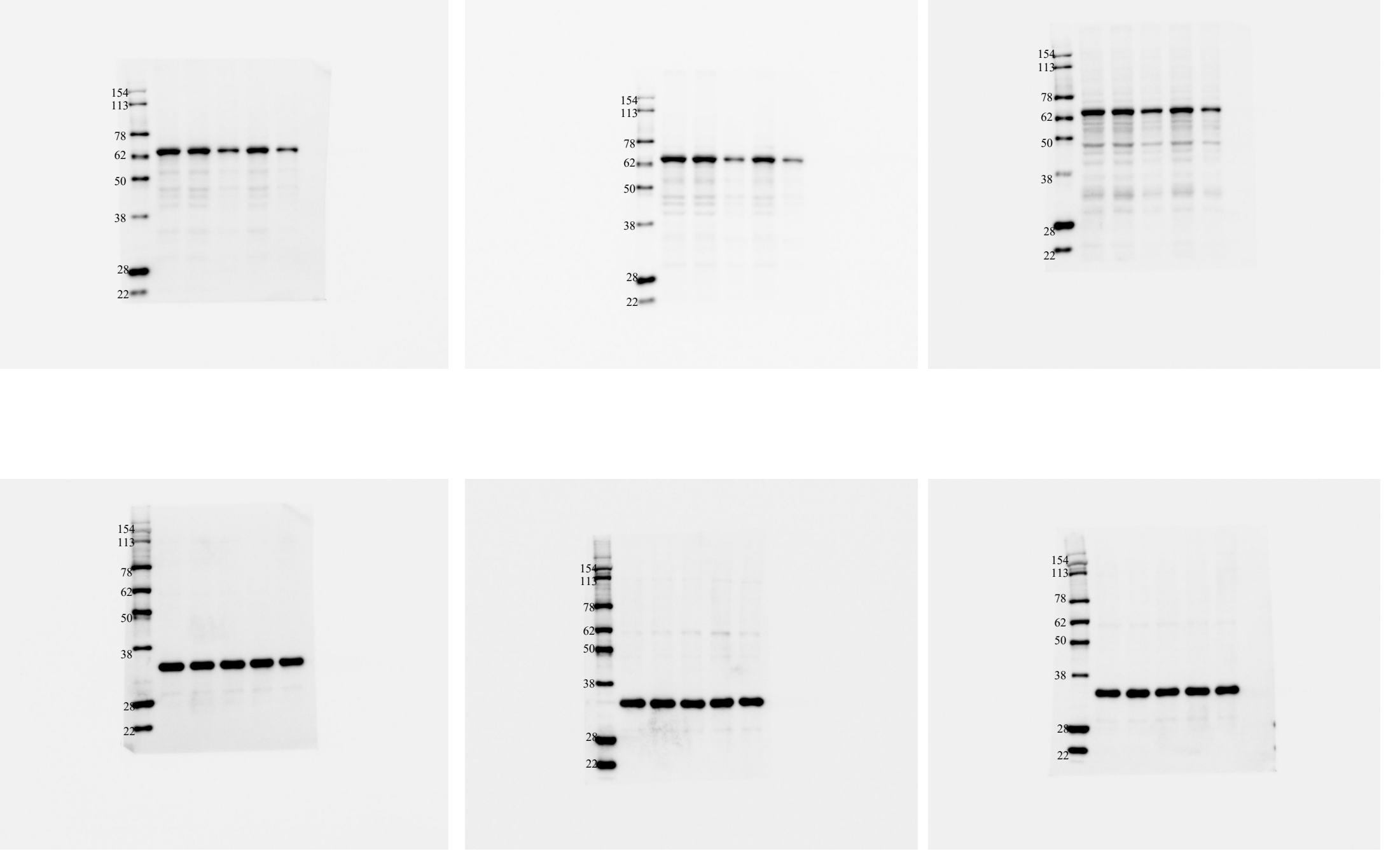

Marker: G2086 Servicebio

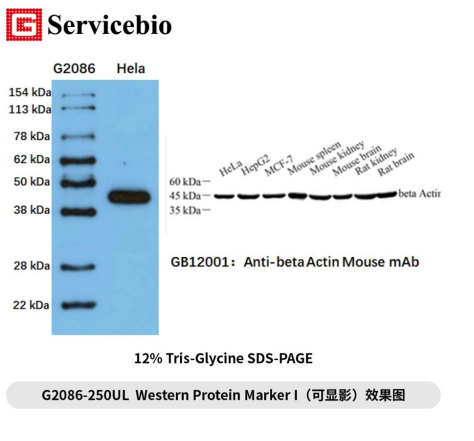

Fig 3-F

1 2 3

p-PI3K
85 kDa

PI3K
85 kDa

p-AKT
60 kDa

AKT
60 kDa

GAPDH
36 kDa

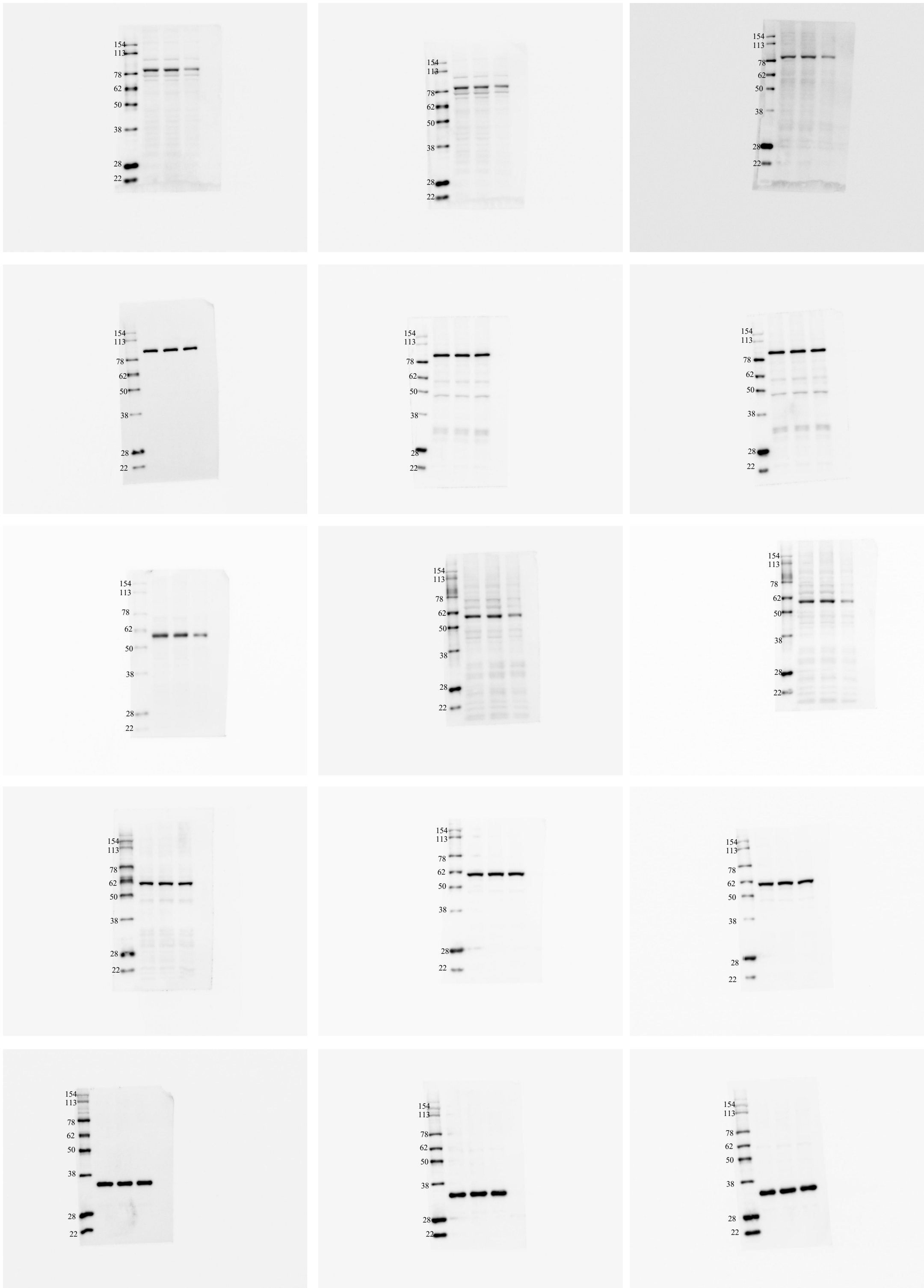

Marker: G2086 Servicebio

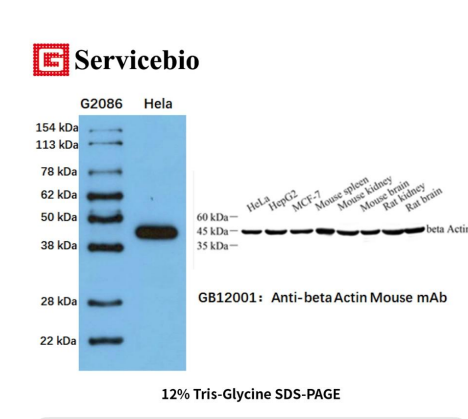

Fig 4-A

	1	2	3

p-PI3K
85 kDa

PI3K
85 kDa

p-AKT
60 kDa

AKT
60 kDa

GAPDH
36 kDa

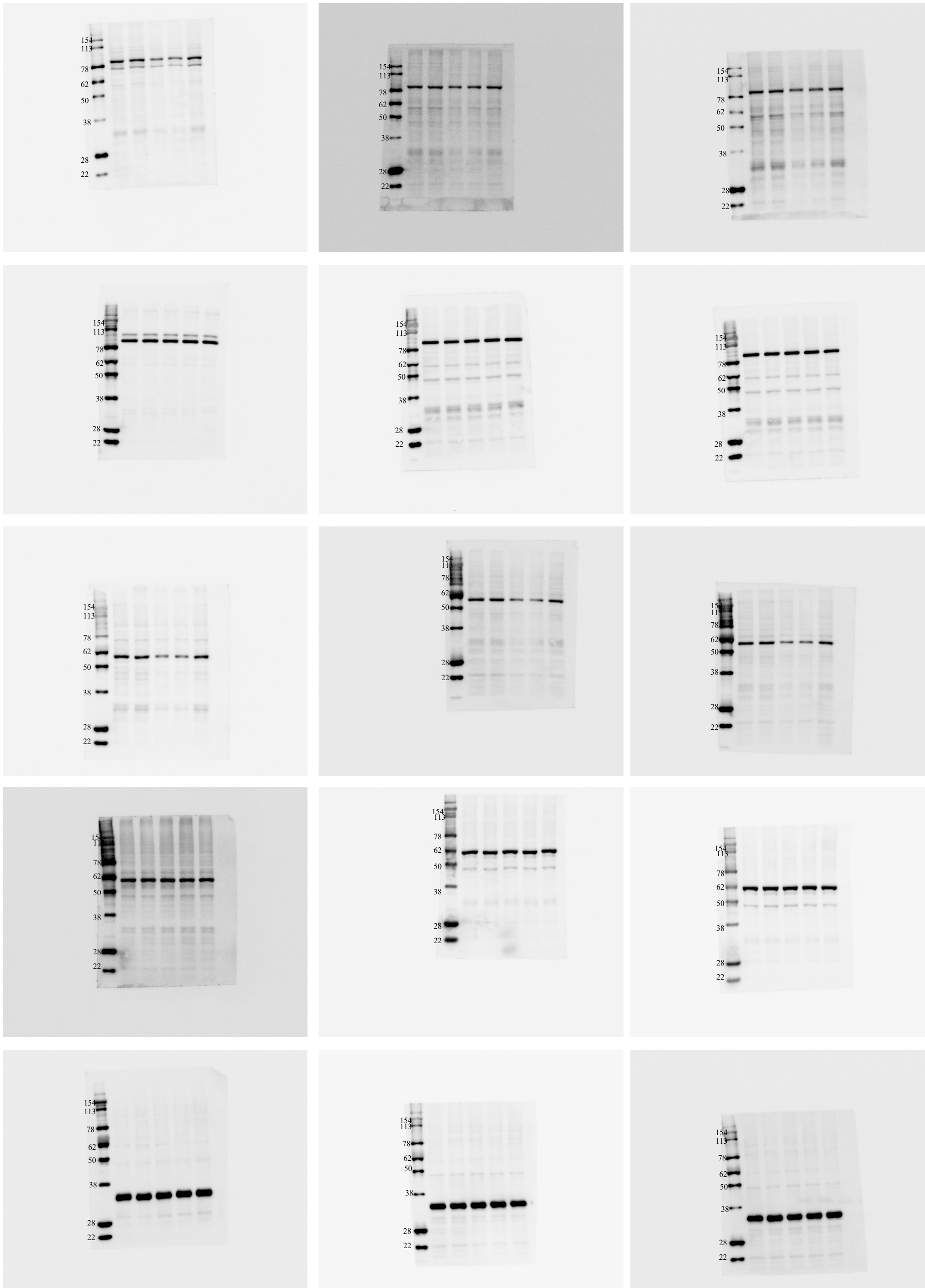

Marker: G2086 Servicebio

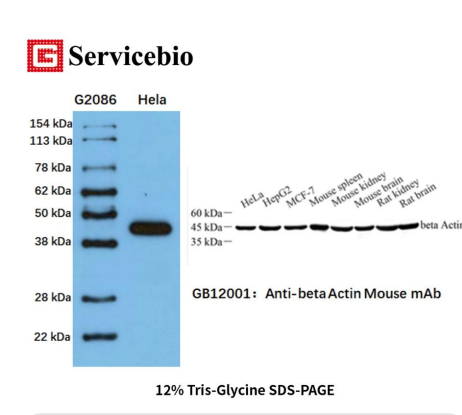

Fig 5-A

	1	2	3
NR4A3<br>65 kDa			
GAPDH<br>36 kDa			

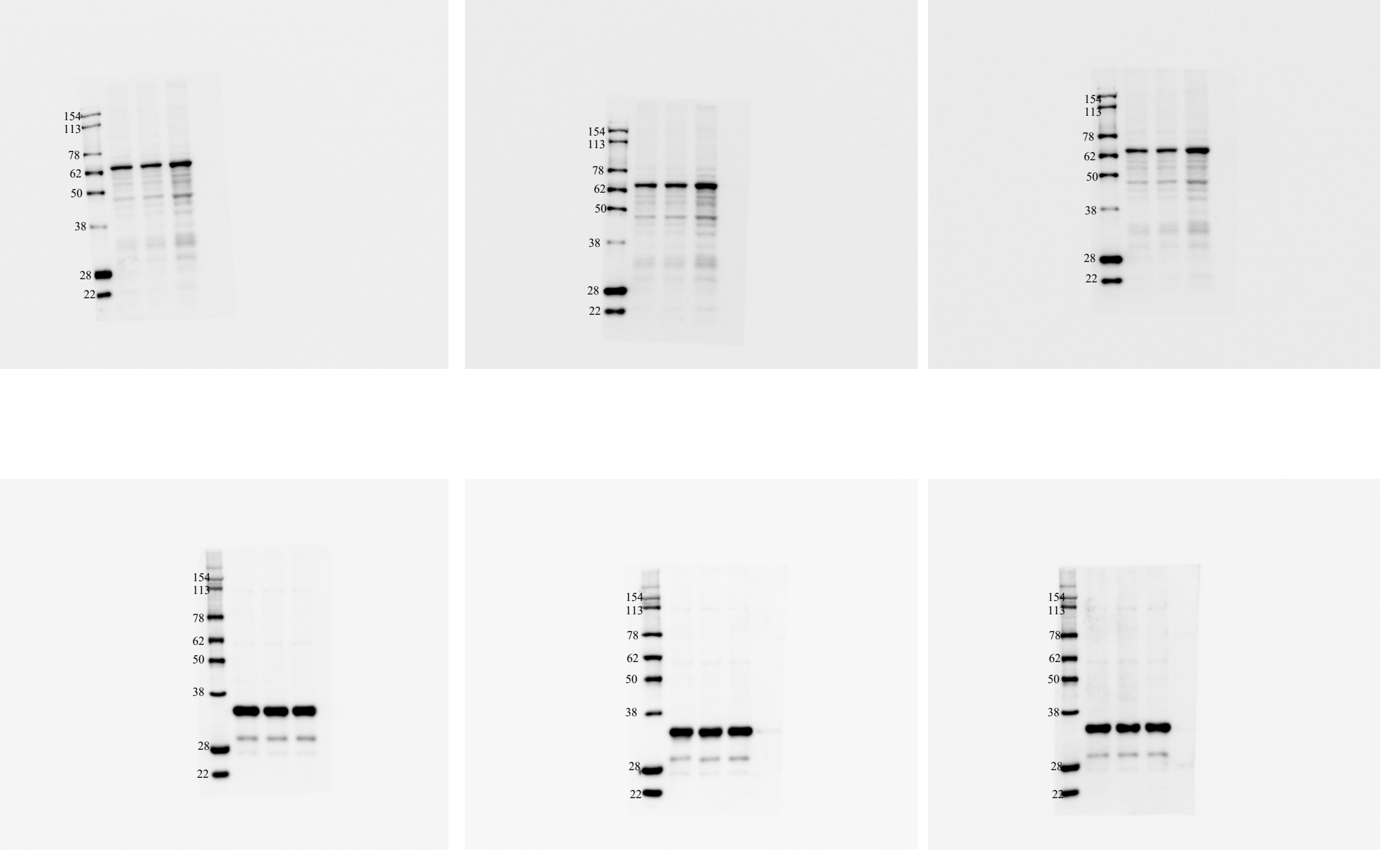

Marker: G2086 Servicebio

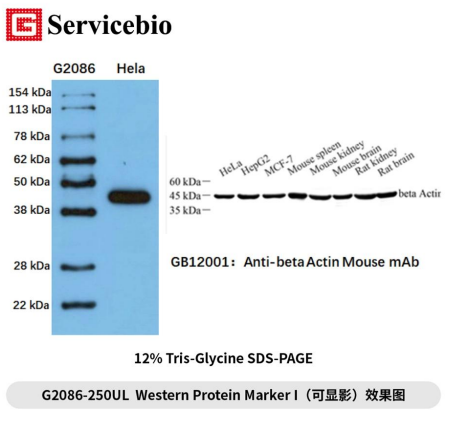

Fig 5-G

1 2 3

p-PI3K 85 kDa

PI3K 85 kDa

p-AKT 60 kDa

AKT 60 kDa

GAPDH 36 kDa

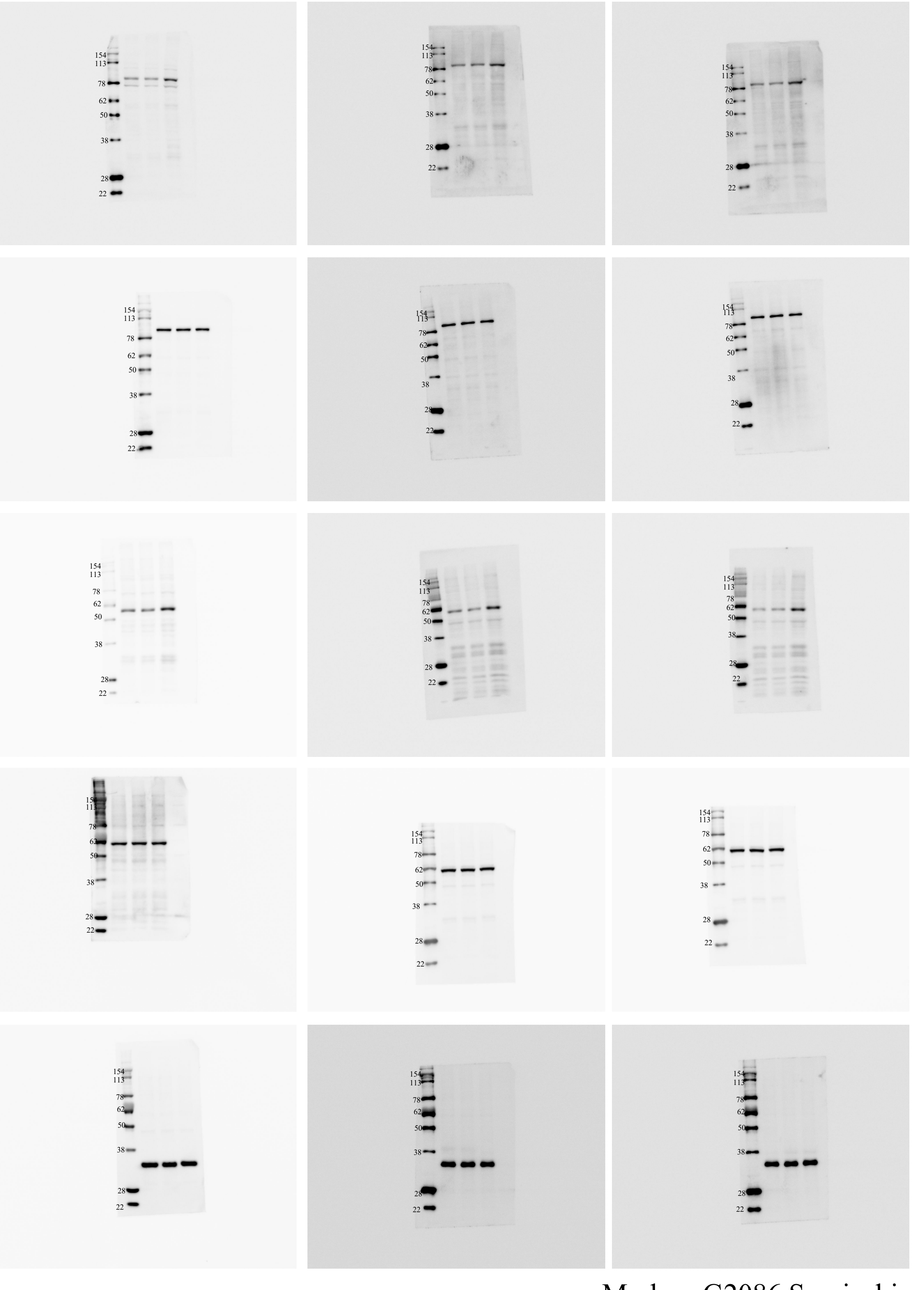

Marker: G2086 Servicebio

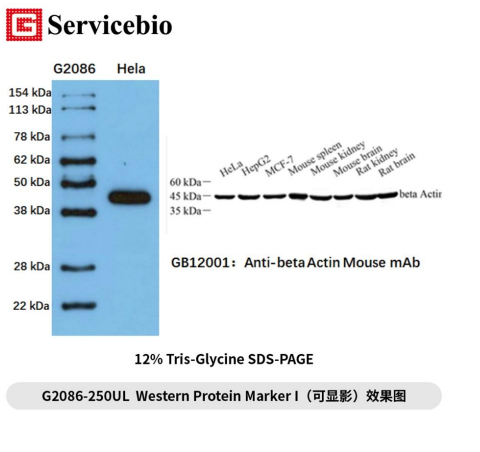

Fig 6

1 2 3

p-GSK3β
46 kDa

GSK3β
46 kDa

p-mTOR
289 kDa

mTOR
289 kDa

GAPDH
36 kDa

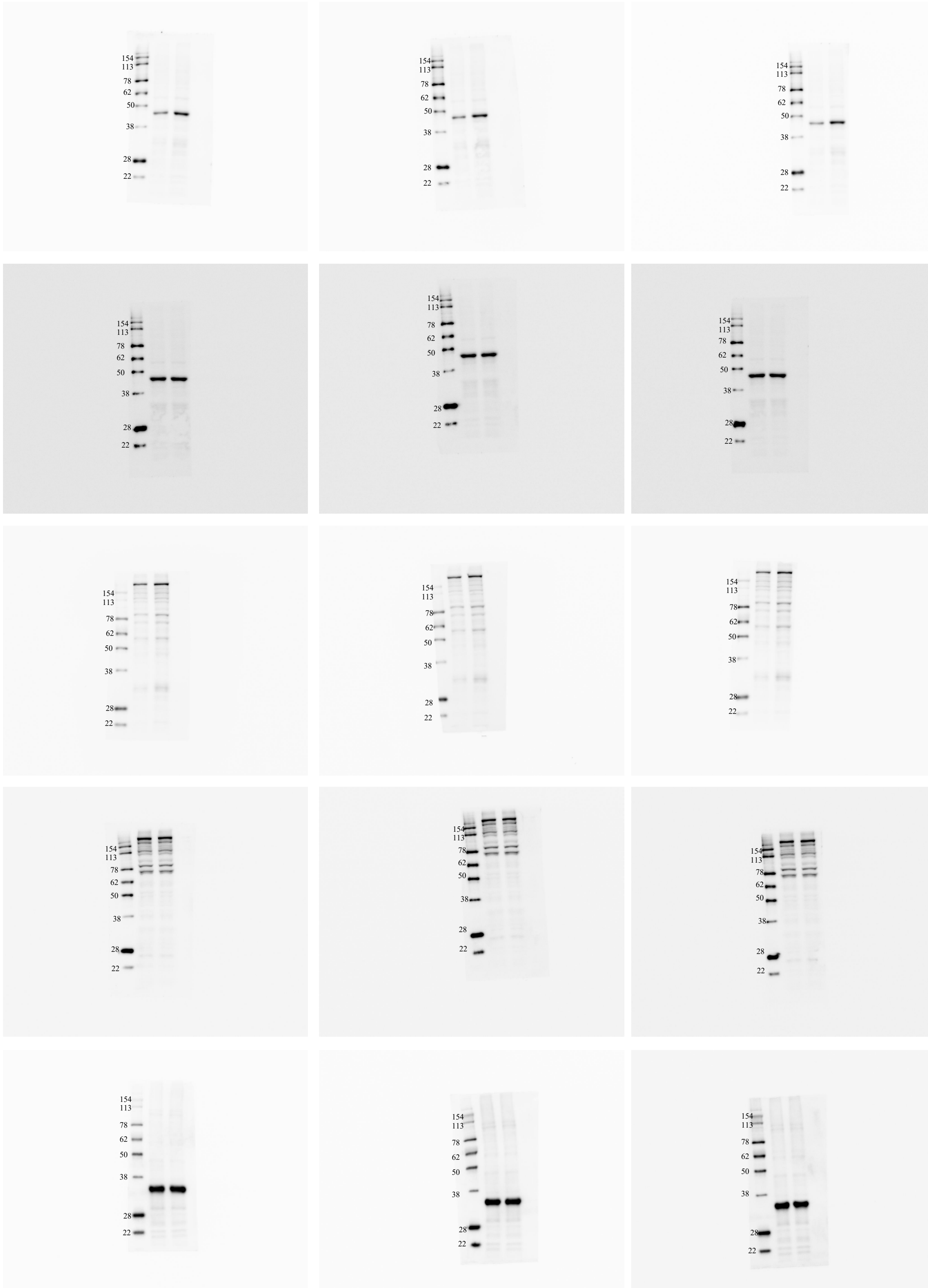

Marker: G2086 Servicebio

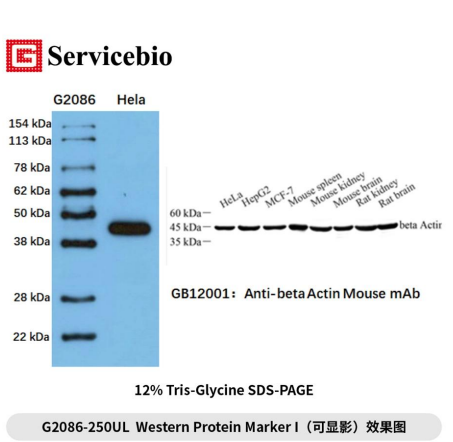

Supplement: Supplementary file 1 [file DataSheet1.pdf]
